# Short palate, lung, and nasal epithelial clone 1 (SPLUNC1) level determines steroid-resistant airway inflammation in aging

**DOI:** 10.1152/ajplung.00315.2021

**Published:** 2021-12-01

**Authors:** Anil Kumar Jaiswal, Jyoti Yadav, Sangeet Makhija, Maninder Sandey, Amol Suryawanshi, Amit Kumar Mitra, Amarjit Mishra

**Affiliations:** ^1^Laboratory of Lung Inflammation, College of Veterinary Medicine, Auburn University, Auburn, Alabama; ^2^Department of Pathobiology, College of Veterinary Medicine, Auburn University, Auburn, Alabama; ^3^Department of Drug Discovery and Development, Harrison School of Pharmacy, Auburn University, Auburn, Alabama; ^4^Center for Pharmacogenomics and Single-Cell Omics, Harrison School of Pharmacy, Auburn University, Auburn, Alabama

**Keywords:** aging, airway inflammation, dendritic cells, SPLUNC1, steroid-resistant asthma, INTRODUCTION

## Abstract

Asthma and its heterogeneity change with age. Increased airspace neutrophil numbers contribute to severe steroid-resistant asthma exacerbation in the elderly, which correlates with the changes seen in adults with asthma. However, whether that resembles the same disease mechanism and pathophysiology in aged and adults is poorly understood. Here, we sought to address the underlying molecular mechanism of steroid-resistant airway inflammation development and response to corticosteroid (Dex) therapy in aged mice. To study the changes in inflammatory mechanism, we used a clinically relevant treatment model of house-dust mite (HDM)-induced allergic asthma and investigated lung adaptive immune response in adult (20–22 wk old) and aged (80–82 wk old) mice. Our result indicates an age-dependent increase in airway hyperresponsiveness (AHR), mixed granulomatous airway inflammation comprising eosinophils and neutrophils, and Th1/Th17 immune response with progressive decrease in frequencies and numbers of HDM-bearing dendritic cells (DC) accumulation in the draining lymph node (DLn) of aged mice as compared with adult mice. RNA-Seq experiments of the aged lung revealed short palate, lung, and nasal epithelial clone 1 (SPLUNC1) as one of the steroid-responsive genes, which progressively declined with age and further by HDM-induced inflammation. Moreover, we found increased glycolytic reprogramming, maturation/activation of DCs, the proliferation of OT-II cells, and Th2 cytokine secretion with recombinant SPLUNC1 (rSPLUNC1) treatment. Our results indicate a novel immunomodulatory role of SPLUNC1 regulating metabolic adaptation/maturation of DC. An age-dependent decline in the SPLUNC1 level may be involved in developing steroid-resistant airway inflammation and asthma heterogeneity.

Asthma is a chronic disease of the upper airways that influence people differently across the lifespan, affecting 7% of the population older than 65 yr (1–3). The heterogeneity of asthma is known to change with age and complicate responses to therapy, thereby impacting health outcomes (4, 5). Asthma in older adults is superimposed on a background of aging-associated lung and immune changes and classified as long-standing (childhood-onset) or late-onset asthma (6–9). A large body of evidence from human studies and rodent models implicates that airway inflammation associated with asthma and responses to therapy in the elderly differs from that in adult subjects with asthma (9–12). Neutrophilic airway inflammation, similar to that noted in severe noneosinophilic asthma, is present in the airways of older people and responds poorly to steroid therapy (7, 13, 14). Increased airspace neutrophil numbers contribute to asthma exacerbation in the elderly, mirroring the changes seen in patients with severe asthma with neutrophil-predominant and mixed Th1/Th17 cytokine signatures (3, 15–17). However, little is known whether this heterogeneity resembles the same disease in aged individuals as in adults or at least has a different cause and pathophysiology.

The microenvironment within the airways and bronchioles changes during aging and airway inflammation. For example, senescence-associated secretory phenotype (SASP) components wield potent effects on adjacent cells and influence dendritic cell-airway epithelial cell (DC-AEC) cross talk and T cell effector function, each of which regulates adaptive lung immunity ultimately promoting functional airway deterioration (18–23). The airway mucosa is lined by AECs, closely associated with antigen-presenting DCs, and secretes inflammatory mediators, activating the local DC network in the airways (24, 25). Subsequently, the development of an effective T cell-mediated adaptive immune response to inhaled allergens requires activation and efficient allergen recognition by DCs (26, 27). Current geriatric studies suggest that DCs from aged individuals are more proinflammatory and exist in a semiactivated state in the absence of stimuli than DC from adults, which triggers the AECs to secrete chemokines altering the barrier properties (28, 29). Moreover, aged DC are less responsive to retinoic acid (RA), impair mucosal tolerance, and accelerate steroid-resistant airway inflammation (30–32). However, changes in airway signals and molecular mechanisms that impact lung DC function and consequently modify the adaptive immune response to the inhaled allergen, triggering asthma in aging, are poorly understood.

Short palate, lung, and nasal epithelial clone1 (SPLUNC1) are the most abundantly expressed secretory proteins in healthy lungs, which serve as an allosteric modulator of epithelial Na^+^ channel (ENaC), regulates mucociliary clearance, and maintains epithelial integrity in the upper airways (33–36). Moreover, due to its structural similarity with other bacterial permeability-increasing protein fold-containing (BPIF) family members and LPS-binding capacity, antimicrobial functions against various pathogens have been suggested (37–40). Previous studies have demonstrated that the production of SPLUNC1 by epithelial cells is markedly reduced in Th1-induced or Th2-induced airway inflammation, suggesting a direct role of SPLUNC1 in allergic asthma (38, 41). Interestingly, in an allergic setting, it has been shown that *SPLUNC1*^−/−^ mice develop severe eosinophilic airway inflammation due to the increased capacity of eotaxin-2 production by alveolar macrophages (42). Furthermore, SPLUNC1 acts as a smooth muscle relaxing factor and suppresses smooth muscle contractility by directly binding and inhibiting Ca^2+^ influx channel Orai1 (43). Although the ion-channel regulating function and the antimicrobial role of SPLUNC1 are known, the biological significance of an immunomodulatory function remains unclear.

In the present study, we show that the response to common aeroallergen house-dust mite (HDM) and induction of airway inflammation is dysregulated with increasing age, which is manifested by steroid-resistant neutrophilic inflammation and Th1/Th17 signatures in the aged lung as compared with eosinophilic/Th2 high adult lung. We demonstrate that epithelium-derived SPLUNC1 levels in the airways progressively decline with increasing age and further by inflammation. Ex vivo, we show that recombinant (r) SPLUNC1 accelerates DC glycolytic reprogramming, maturation, and promotes proliferation of OTII and Th2 cytokine secretion. Our findings collectively suggest SPLUNC1 as a steroid-responsive gene promoting DC-mediated immune priming function that progressively declines with age. Therefore, we propose that a reduction in SPLUNC1 level may in part accelerate steroid-resistant airway inflammation and asthma heterogeneity in aging.

## METHODS

The authors declare that all supporting data are available within the article. All procedures and protocols involving animals were approved by the Institutional Animal Care and Use Committee of Auburn University (Auburn, AL).

### Mice Allergen (HDM) Sensitization/Challenge and Dex Treatment Protocol

Young (6–8 wk old), adult (20–22 wk old), and aged (80–82 wk old) wild-type BL/6J mice of both sexes and B6.Cg-Tg(Tcra Tcrb)425Cbn/J, which expresses a transgenic MHCII-restricted TCR that binds the OVA peptide antigen, OVA_323–339_ mice (44) were purchased from Jackson Laboratories (Bar Harbor, ME). Mice were sensitized with 50 μg of HDM in a volume of 40 μL through the intranasal (in) route on *days 1*, *3*, and *5*. The challenge phase was divided into three consecutive HDM administration phases (50 μg in administration/mouse) on *days 11–13*, *18–20*, and *25–27*, respectively. In the treatment protocol, mice receive dexamethasone injection (Sparhawk Laboratories, Lenexa, KS) (4 mg/kg body wt, ip) or sterile saline every 72 h during the HDM challenge period. After the last HDM challenge, mice were anesthetized using ketamine (100 mg/kg) and xylazine (10 mg/kg) and bronchoalveolar lavage (BAL), mediastinal lymph node (DLn), and lung lobes were harvested and analyzed.

### Measurement of Airway Hyperresponsiveness

The trachea was cannulated with a 19-G beveled metal catheter, and airway resistance to increasing concentrations of methacholine (Acetyl β-methyl choline, Sigma Aldrich, St. Louis, MO) was directly measured in mechanically ventilated anesthetized mice using an Elan RC Fine Pointe system (DSI, St. Paul, MN). A nebulizer was used to administer ascending doses of methacholine (0, 2.5, 5.0, and 10 mg/mL), and means ± SE values are presented as cmH_2_O per mL/s.

### Assessment of Airway Inflammation

To quantify the inflammation of the airways, each mice lung was washed three times with 0.5 mL of DPBS. Furthermore, RBCs were lysed with ACK lysis buffer for 2–3 min at 4°C, and cells were resuspended in RPMI medium with 10% FBS. BAL cell counts were performed using a hemocytometer, and differential staining for infiltrating cells was performed by flow cytometry. BAL levels of CCL24 and CXCL1 (KC1) chemokines were determined using quantitative ELISA kits from R& D Systems (Minneapolis, MN). The lung was fixed with 10% formalin for 24 h, dehydrated through gradient ethanol, and embedded in paraffin for histopathology. Lung sagittal sections were cut to a thickness of 5 μm and stained with hematoxylin and eosin.

### In Vivo Dendritic Cell Migration

HDM extracts (*Dermatophagoides pteronyssinus*) were labeled with Alexa Fluor 647 (AF647) Protein Labeling Kit (Molecular Probes, Life Technologies) according to the manufacturer’s instruction. The labeled HDM was administered (50 µL; in administration) to adult (20–22 wk old) and aged (80–82 wk old) mice. Lungs and DLNs were harvested after 72 h, and the number of CD11c^+/^MHC-II^hi/^SSC^lo/^CD11b^+/^HDM^+^ DCs were quantified by flow cytometry as reported before (45).

### Flow Cytometry

Lungs lobes were harvested and digested by enzymatic digestion using type IV collagenase (1 mg/mL) and DNase I (0.1 mg/mL) (Worthington, Lakewood, NJ) for 30 min at 37°C with agitation. Before performing surface staining, suspensions of single cell (1–2 × 10^6^ cells) were preincubated with Fc Block antibody (BD PharMingen) in staining buffer [1X DPBS, 3% FBS, 2 mM EDTA, and 10 mM 4-(2-hydroxyethyl)-1-piperazineethanesulfonic acid] at 4°C for 10 min. Furthermore, cells were first stained with Live/Dead Fixable Violet Staining Kit (Invitrogen) in 50 μL of PBS for 15 min at 4°C. After washing cells with staining buffer, cocktails of desired cell surface antigens were incubated in 50 μL of staining buffer for 30 min at 4°C in the dark. BAL eosinophils (Eos), neutrophils (Neu), macrophages (Mac), and lymphocytes (Lym) were identified using antibodies against rat anti-mouse CD45R(B220)-Alexa Fluor 488 (clone, RA3-6B2), CD3-Alexa Fluor 488 (clone, 145-2C11), I-A/I-E (MHC-II)-PE-Cy7 (clone, M5/114.15.2), CD11c-APC-eFluor780 (clone, N418), and F4/80-E-Fluor 450 (clone, BM5) all from eBiosciences except anti-mouse CCR3-PE (clone 83101) (R&D Systems) and Gr1-(Ly6G/Ly6C)-APC (clone, RB6-8C5) from BioLegend. To detect Glut-1 expression, cells were stained with live/dead fixable stain and immediately fixed with Fix/Perm buffer (eBiosciences). After washing, cells were stained with anti-Glut-1-PE antibody (Novus biologicals), and surface and endogenous expressions of Glut-1 were determined by flow cytometry.

To assess the intracellular cytokines, single-cell lung suspensions were first stained with surface markers against rat anti-mouse CD3-Alexa Fluor 647 (clone 17-A2), CD4-APC-eFluor 780 (clone GK1.5) from eBiosciences. After a single washing with staining buffer, cells were further fixed for 30 min with IC Fixation Buffer (eBiosciences). Followed by a wash with 1X Perm/Wash buffer (BD Biosciences), cytokines were detected after incubation with monoclonal antibody cocktails of rat anti-mouse IL-13-Alexa Fluor 488 (eBio13A), IL-17-eFluor 450 (eBio17B7), and IFN-γ-PerCP-Cy5.5 (cone, XMG1.2), (all from eBioscience) in 50 μL 1X Perm/Wash buffer (BD Biosciences) for 30 min at room temperature. Cells were washed and resuspended in staining buffer and analyzed by flow cytometry. Absolute cell counts were determined using hemocytometer, and flow cytometry data were acquired on a cytometer (LSR-II; BD Biosciences). Data were further analyzed using FlowJo software (TreeStar).

### Magnetic Sorting of Lung Dendritic Cells and Airway Epithelial Cells

Single-cell suspension was prepared using freshly isolated mice lungs, as stated earlier. Airway epithelial cells (AECs) were isolated with EasySep APC Positive Selection Kit (StemCell Technologies, UK) according to the manufacturer’s instructions in 5-mL polystyrene round-bottom tubes on the EasySep magnet. Cell suspensions (1 × 10^8^ cells/mL) were centrifuged at 300 *g* for 10 min at 4°C and resuspended in 0.5 mL of recommended medium (PBS containing 2% FBS and 1 mM EDTA) in a 5-mL polystyrene tube. Furthermore, mouse-specific FcR block (1 µg/mL) was added and stained with epithelial cell-specific CD326 (EpCAM) monoclonal antibody (1 µg/mL; clone: G8.8, APC, eBioscience). Using selection cocktail and Rapid Spheres, EpCAM+ cells were selected and were processed for further DC isolation (EasySep Mouse Pan-DC Enrichment Kit; StemCell Technologies) according to the manufacturer’s instructions. Positively selected lung AECs and negatively sorted lung DCs were processed for RNA isolation.

### Quantitative Real-Time Polymerase Chain Reaction

RNA from lung tissue and isolated DCs, AECs, or cultured BMDCs were isolated using TRIzol reagent (Life Technologies, Grand Island, NY), and cDNA was prepared with a High-Capacity RNA to cDNA kit (Applied Biosystems). Quantitative real-time polymerase chain reaction (qRTPCR) was performed using the QuantStudio 6 sand 7 Flex Real-Time PCR Systems (Applied Biosystems). All the genes were quantified using SYBR green primers (Table 1). After amplification, C_t_ values were obtained and analyzed according to the 2^−ΔΔCt^ method considering quantitative PCR efficiency. The C_t_ value of each target gene was normalized to housekeeping gene acidic ribosomal phosphoprotein P0 (36B4).

**Table 1.** List of primer sequences used in the study

	Forward Primer	Reverse Primer
*BPIFA1*	5′- GTCCACCCTTGCCACTGAACCA-3′	5′- CACCGCTGAGAGCATCTGTGAA-3′
*TGF-β*	5′- GGATGCATTCATGAGTATTGC-3′	5′- GCTTCCTGAGGCTGGATTC-3′
*Col1*	5′- TGGACGCCATCAAGGTCTACTGC-3′	5′- GGAGGTCTTGGTGGTTTTGTATTCG-3′
*MMP10*	5′- CCTGTGTTGTCTGTCTCTCCAAGA-3′	5′- CGTGCTGACTGAATCAAAGGAC-3′
*MMP12*	5′- AATTACACTCCGGACATGAAGCGT-3′	5′- GGCTAGTGTACCACCTTTGCCATC-3′
*GR1-α*	5′- AAAGAGCTAGGAAAAGCCATTGTC-3′	5′- TCAGCTAACATCTCTGGGAATTCA-3′
*GR1-β*	5′- AAAGAGCTAGGAAAAGCCATTGTC-3′	5′- CTGTCTTTGGGCTTTTGAGATAGG-3′
*PKM2*	5′- TTAGGCCAGCAACGCTTGTAGTGC-3′	5′- AGATGCTGCCGCCCTTCTGTGATA-3′
*Hif-1α*	5′- GGTTCCAGCAGACCCAGTTA-3′	5′- AGGCTCCTTGGATGAGCTTT-3′
*HK2*	5′- TGATCGCCTGCTTATTCACGG-3′	5′- AACCGCCTAGAAATCTCCAGA-3′
*IRF4*	5′- ACAGCACCTTATGGCTCTCTG-3′	5′- ATGGGGTGGCATCAT GTAGT-3’
*ICOSL*	5′- AGCTTGAACTTACAGACCACGC-3′	5′- CTCTGAAGTTGTGTCTGACATC-3′
*Zbtb46*	5′- AGAGAGCACATGAAGCGACA-3′	5′- CTGGCTGCAGACATGAACAC-3′
*OX40L*	5′- ATGGAAGGGGAAGGGGTTCAACC-3′	5′- TCACAGTGGTACTTGGTTCACAG-3′
*Glut1*	5′- CATCCTTATTGCCCAGGTGTTT-3′	5′- GAAGACGACACTGAGCAGCAGA-3′
*LDHA*	5′- CACTGACTCCTGAGGAAGAGGCCC-3′	5′- AGCTCAGACGAGAAGGGTGTGGTC-3′
*Zbtb46*	5′- AGAGAGCACATGAAGCGACA-3′	5′- CTGGCTGCAGACATGAACAC-3′
*36B4*	5′- GGACCCGAGAAGACCTCCTT-3′	5′- GCACATCACTCAGAATTTCAATGG-3′

p16 (INK4a) (Cdkn2a): TaqMan primer; assay ID: Mm00494449_m1 (Invitrogen: Cat No: 4453320).

### Flow Cytometric Detection of SA-β-Galactosidase

SA-β-galactosidase was detected by flow cytometry in the lung CD45^−^/EpCAM^+^ epithelial cells (AECs), CD45^−^/CD31^+^ endothelial cells (Endo), and CD45^+^/CD11c^+^/CD11b^+^/MHCII^+^ dendritic cells (DC). In brief, single-cell lung suspensions from adult and aged mice were obtained by enzymatic digestion as described above. Lung cell suspensions were incubated for 2 h with C12FDG (33 µM, Thermo Fischer) followed by washing with ice-cold PBS before fluorescence measurement using a flow cytometer (LSR-II, BD Bioscience). Data were analyzed using the FlowJo software.

### Bone Marrow-Derived Dendritic Cell Cultures and Stimulation

Bone marrow-derived dendritic cells (BMDCs) were generated as previously described (46, 47). Briefly, cells were isolated from leg bones of euthanized mice and cultured at a density of 1 × 10^6^ cells/mL in differentiation media [Iscove modified Dulbecco medium (Gibco/Life Technologies, CA) containing 10% heat-inactivated FCS, penicillin-streptomycin (100 U/mL), L-glutamine (2 mM), 2-mercaptoethanol (50 µM), recombinant mouse granulocytes macrophage colony-stimulating factor (20 ng/mL), and recombinant mouse IL-4 (10 ng/mL, PeproTech]. Cultures were supplemented with an equal volume of medium (*day 3* and *day 5*), and nonadherent cells were collected on *day 6* and plated in six-well tissue culture plates at a density of 2 ×10^6^ cells/well in differentiation media. Nonadherent cells were collected on *day 7* for analysis. BMDCs (2−5 × 10^5^) were cultured on 96-well plates and pulsed with HDM (100 µg/mL) in the presence or absence of rSPLUNC1 (5 µg/mL). BMDCs were further processed for RNA isolation and Western blot analysis. In separate experiments, BMDCs were used for XFp assays and extracellular acidification rates (ECARs) measurements.

### BMDC and OT-II Coculture

The ability of BMDCs to induce ex vivo antigen-specific T cell proliferation was assessed using CFSE-labeled splenic CD4^+^ T cells obtained from naïve [B6.Cg-Tg(Tcra Tcrb)425Cbn/J] transgenic mice (Jackson Laboratories, Bar Harbor, ME) that express a transgenic MHCII-restricted TCR that recognizes the OVA peptide antigen (44). Splenic naive CD4^+^ T cells were purified using EasySep Mouse CD4^+^ T Cell Isolation Kit (Stem cells, Vancouver, CA) and were labeled with 5 µM CFDA-SE (carboxyfluorescein diacetate succinimidyl ester; Cayman Chemical, MI) in DPBS for 20 min at 37°C. BMDCs were pulsed overnight with 5 µg·mL^−1^ of OVA_323–339_ peptide (AnaSpec, Fremont, CA) or PBS in the presence or absence of rSPLUNC1 (5 µg/mL. Furthermore, 1 × 10^5^ OVA peptide-specific CD4^+^ OT-II cells were cocultured with 2 × 10^4^ CD11c^+^ BMDCs in 96-well plates for 4 days, and T cell proliferation was quantified by flow cytometry using CFSE dye dilution. Gated CD4^+^ T cells were analyzed using proliferation profile and percent divided as calculated by the FlowJo Proliferation Platform. The quantity of IL4 and IL13 released into the culture supernatant were measured using ELISA. In addition, cells were stained with CD3-Alexa Fluor 647 (clone 17-A2), CD4-APC-Cy7 780 (clone GK1.5) before incubation with GATA3-BV405 (clone TWAJ) for flow cytometry.

### ELISA and Immunoblot Assay for SPLUNC1 Detection

ELISA plates (Nunc MaxiSorp) were coated with rSPLUNC1 and BAL samples and further incubated with polyclonal sheep anti-mouse SPLUNC1 antibody (R&D, Minneapolis, MN), Sheep IgG horseradish peroxidase (HRP) (R&D, Minneapolis, MN), and tetramethylbenzidine substrate (Thermo Fisher Scientific). Data were measured at an optical density of 450 and 560 nm after background correction. To quantify SPLUNC1 level in BAL samples, an equal amount of BAL were resolved by SDS-PAGE using 4%–12% Bis-Tris gel (Thermo Fisher Scientific) and transferred on nitrocellulose membranes (Thermo Fisher Scientific) and probed with anti-mouse SPLUNC1 (1:500; R&D Systems) and anti-rabbit IgG, HRP-linked antibody (1:2,000; Cell Signaling Technologies).

### RNA Sequencing Experiments and Analysis

Total RNA from lung samples of the adult (20–22 wk old) and aged (80–82 wk old) naïve, HDM-treated, and HDM + Dex-treated (*n* = 5) were extracted using RNeasy plus Universal Kits (QIAGEN, MD). The quality of RNA was assessed before being processed for library preparation using Bioanalyzer (Agilent Technologies). The whole transcriptome was amplified, and the library was constructed using TruSeq Stranded mRNA (Illumina; San Diego, CA). Quantitative assessment of library was done using Qubit 2.0 fluorometer (Invitrogen) and evaluated on the high-sensitivity DNA chip (Agilent Technologies). Libraries were sequenced on a NovaSeq 6000 platform (Illumina) using a pair-end 50 bp sequencing strategy. RNA sequencing data were preprocessed and analyzed using the Picard-STAR-limma pipeline. Raw read counts and fragments per kilobase of transcript per million (FPKM) mapped reads abundance were estimated at the transcript and gene levels. Principal component analysis (PCA) was used to identify outliers. Both upregulated and downregulated gene sets are reported on three GO subcategories: biological process (BP), cellular component (CC), and molecular function (MF). FDR q-values were estimated to correct the *P*-values for the multiple testing issue. The data discussed in this publication have been deposited in NCBI’s Gene Expression Omnibus and are accessible through GEO Series accession number GSE178770 (https://www.ncbi.nlm.nih.gov/geo/query/acc.cgi?acc=GSE178770).

### Seahorse Analysis

The Seahorse XFp Extracellular Flux Analyzer (Agilent, Santa Clara, CA) was used to analyze real-time changes of extracellular acidification rates (ECARs) as described earlier (48). BMDCs were plated (120,000 cells/well) in 200 µL in XFp mini-culture plates overnight in an IMDM medium containing 5% FBS at 37°C in the CO_2_ incubator. The culture medium was replaced the next day with a fresh medium containing 5% FCS, stimulated with HDM (100 µg/mL), and treated with and without rSPLUNC1 protein for 16 h. Furthermore, the medium was replaced with a warm X_F_ DMEM base medium (Agilent) containing 2 mM L-glutamine (Agilent, 103579) and 1% FBS. The assay plate was incubated in a non-CO_2_ incubator for 1 h at 37°C. Glycolysis stress tests were performed according to the manufacturer’s protocol with sequential injections of glucose, oligomycin (Agilent), and 2-DG (Sigma) in ports A, B, and C, respectively. The effects on ECAR were recorded three times every 5 min interval, and data were analyzed using Wave software 2.6.1 (Seahorse Bioscience) after normalization with total protein.

### Statistics

Data were analyzed using GraphPad Prism version 7.0 b using either an unpaired *t* test for normally distributed data or a Mann–Whitney test for non-normally distributed data. Multiple comparisons were analyzed using a one-way ANOVA with Sidak’s multiple comparison test or a two-way ANOVA. Data are presented as means + SE. A *P* value < 0.05 was considered significant.

## RESULTS

### Age-Dependent Increase in Steroid-Resistant Neutrophilic Airway Inflammation

**Figure 1.**
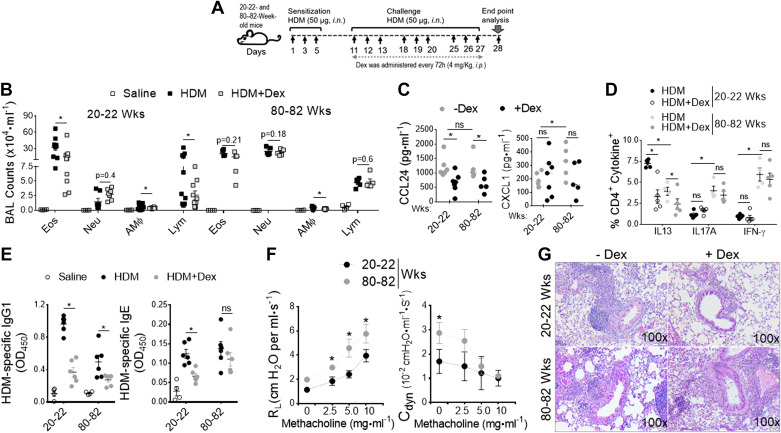
Aging promotes Dex-resistant mixed granulomatous airway inflammation. *A*: adult (20–22 wk old) and aged (80-82 wk old) B6 mice were intranasally sensitized and challenged with HDM (50 μg) and PBS. Mice received Dex (4 mg/kg body wt, ip) or sterile saline every 72 h during the HDM-challenged period as indicated. Endpoint analysis was performed 24 h after the last administration of HDM. *B*: numbers of BAL inflammatory cell types (Eos; eosinophils), (Neu; neutrophils), (AM; alveolar macrophages), and (Lym; lymphocytes) from PBS^−^, HDM^−^, and HDM^+^ Dex-challenged were compared; (*n* = 8–10 mice, significance denoted by *P< 0.01, HDM vs. HDM+ Dex, one-way ANOVA with Sidak’s multiple comparison test). Bar graph shows (*C*) BAL levels of CCL24 and CXCL1 chemokines; (*D*) frequency of CD4^+^ cytokines^+^ T cells in lung; and (*E*) serum levels of HDM specific IgG1 and HDM-specific IgE (*n* = 8–10 mice, significance denoted by *P < 0.05, HDM vs. HDM^+^ Dex, one-way ANOVA with Sidak’s multiple comparison test). *F*: graph plot show AHR (*left*, airway resistance and *right*, lung dynamic compliance to increasing dose of inhaled methacholine in HDM-treated mice) (*n* = 8–10 mice, significance denoted by *P < 0.05, 20–22 wk old vs. 80–82 wk old, one-way ANOVA with Sidak’s multiple-comparison test), and *G*: representative lung histology sections stained with H&E and PAS. Scale bars = 100 μm for the ×100 images. Data are shown as means ± SE of 5–10 mice per group and representative from three independent experiments. AHR, airway hyperresponsiveness; BAL, bronchoalveolar lavage; HDM, house-dust mite.

Using a clinically relevant murine model of asthma induced by repeated exposure to HDM (*D. pteronyssinus*), a common aeroallergen that is an environmental trigger and risk factor for the development of persistent asthma in the elderly (49), we sought to identify the underlying molecular mechanism of disease severity in the context of aging. We employed a treatment model of HDM-induced asthma in adult (20–22 wk old) and aged (80–82 wk old) B6 mice (Fig. 1*A*). Mice were sensitized and challenged with HDM thrice for three consecutive days with 4 days intervals and received intraperitoneal injections of Dex (4 mg/kg) every 72 h during the challenge period. Differential counts in the bronchoalveolar lavage (BAL) fluid recovered from mice 24 h after the last HDM challenge showed greater neutrophil recruitment in aged mice but more eosinophil predominant airway inflammation in adults (Fig. 1*B*). Dex treatment substantially reduced airway inflammation in adult mice but was ineffective in reducing airway inflammation in the aged. BAL levels of C-C chemokines (CCL24) that recruit eosinophils were significantly decreased with Dex treatment in both ages. However, C-X-C chemokines (KC) that recruit neutrophils were unresponsive to Dex therapy and increased in aged mice compared with adult mice (Fig. 1*C*). In addition, the increase in lung IL13+ CD4+ T cell numbers was greater in adults than in aged mice, and the cells were reduced by 50% upon Dex treatment (Fig. 1*D*). We observed a significantly higher frequency of IL17A^+^ CD4^+^ and IFN-γ^+^ CD4^+^ T cells in aging lungs compared with adults, which was not suppressed by Dex treatment. Although serum levels of HDM-specific IgG1 were significantly decreased with Dex treatment compared with HDM-challenged mice of both ages, HDM-specific IgE levels were not altered in aged mice after Dex treatment (Fig. 1*E*), demonstrating an age-dependent increase in steroid-resistant airway inflammation. Moreover, the aged mice had significantly higher lung resistance and dynamic compliance than HDM-challenged adult mice with increasing doses of methacholine (Fig. 1*F*). In support, histological examinations revealed marked peribronchial inflammatory cell infiltrates in HDM-challenged aged mice, which was not reduced by Dex treatment (Fig. 1*G*). These results demonstrate that the HDM challenge causes predominantly eosinophilic airway inflammation that is fully responsive to Dex treatment in adults, whereas aged lung augments mixed granulomatous neutrophilic inflammation and refractory AHR to Dex treatment.

### A Decline in DC Migration to Draining Mediastinal Lymph Node Is Impacted by Senescence

**Figure 2.**
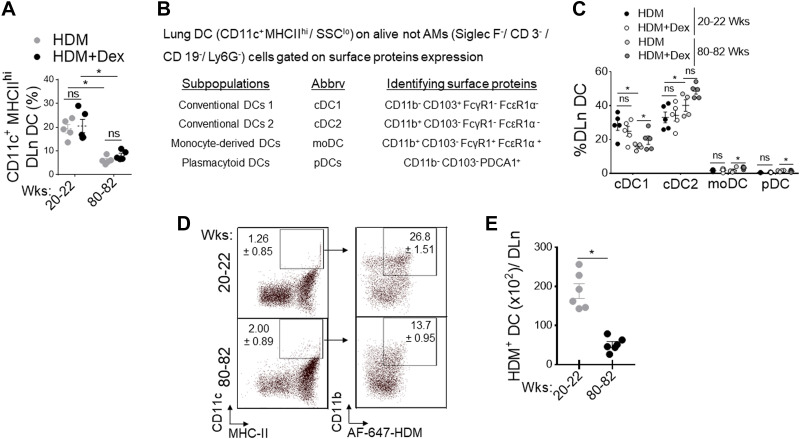
Defective DC migration in aging. Adult (20–22 wk old) and aged (80–82 wk old) wild-type mice were sensitized and challenged by administration of PBS, HDM (50 μg), and or treated with Dex (4 mg/kg, ip). *A*: flowcytometry assessed the frequency of CD11c+ MHC-IIhi CD11b+ DC in DLn (*n* = 4–6 mice per group, significance denoted by *P< 0.01, HDM vs. HDM+ Dex, adult vs. aged, one-way ANOVA with Sidak’s multiple comparison test). *B*: the panel of surface markers used to identify lung DC subsets and (*C*) frequency in adult and aged mice after HDM challenge and Dex treatment (each circle indicates individual mice, *P< 0.01, HDM vs. HDM+ Dex, adult vs. aged, one-way ANOVA with Sidak’s multiple comparison test). Activated DC migration to DLn during the sensitization phase of allergic airway inflammation: Naïve B6 mice intranasally inoculated with AF647-labeled HDM (100 µg/mouse in 50 µL of PBS) and enumerated at 72 h in DLn and analyzed for CD11c+/MHC-II^hi/^CD11b+/HDM+ DC by flow cytometry. *D*: representative dot plot and (*E*) enumeration of migrated AF647+HDM+ DC per DLn. Data are represented as means ± SE of six mice per group and representative of two independent experiments (*P< 0.05, adult vs. aged, unpaired *t* test). DC, dendritic cell; DLn, draining lymph node; HDM, house-dust mite.

**Figure 3.**
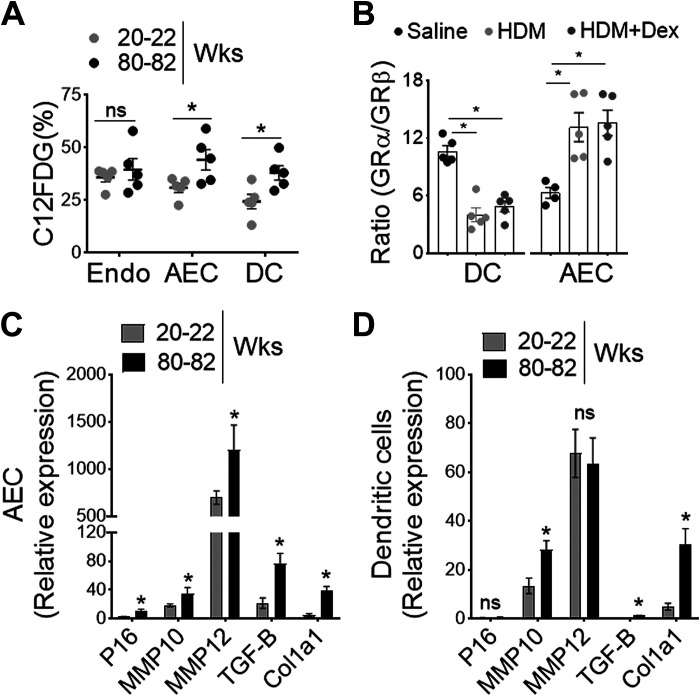
Impact of lung senescence in airway epithelial cells (AECs) and DC. *A*: frequency of C12FDG+ lung endothelial cells (Endo), AECs, and DC from adult and aged mice as measured by SA-β-gal activity-based flow cytometric staining (*n* = 4 or 5 mice per group, *P< 0.01, adult vs. aged, unpaired *t* test), *B*: bar chart shows the ratio of GRα/GRβ expression in isolated lung DCs and AECs from HDM or PBS-challenged aged mice treated with Dex (*n* = 4 or 5 mice per group, *P< 0.01, saline vs. HDM, saline vs. HDM + Dex, one-way ANOVA with Sidak’s multiple comparison test). Bar chart showing relative expression of senescence-associated secretory phenotype (SASP) in isolated (*C*) AECs and (*D*) lung DCs. Data are represented as means ± SE from two independent experiments (*n* = 4 or 5 mice per group, *P< 0.01, adult vs. aged, unpaired *t* test). DC, dendritic cell; DLn, draining lymph node; HDM, house-dust mite; SA-β-gal, SA-β-galactosidase.

Migration of activated tissue DC from the site of inflammation to DLn is an essential step in the induction of lung adaptive immune response to inhaled allergens (27, 46). To investigate whether increasing age influences the lung DC repertoire, we evaluated the numbers of different DC subsets in the HDM-challenged lungs of adult and aged mice with and without Dex treatment. Frequencies of total DC in DLn were markedly reduced in aged mice compared with adult mice and are unresponsive to Dex treatment (Fig. 2*A*). Using a previously described approach (Fig. 2*B*) (45), we found a significant reduction in the cDC1 subset in aged mice. However, the cDC2, moDC, and pDC were comparable in aging and adult mice with HDM challenge and after Dex treatment (Fig. 2*C*). Next, we analyzed the capacity of lung DCs to capture and transport HDM-derived antigens to DLn in response to inhaled HDM extracts after 72 h. We found a progressive decline in frequencies (Fig. 2*D*) and numbers (Fig. 2*E*) of HDM bearing Alexa-647^+^ DC in the DLn as mice aged, suggesting that the intrinsic ability of DC to migrate to DLn is compromised in aged mice. In addition, using C12FDG (5-dodecanoyl amino fluorescein di- β-D-galactopyranoside), a fluorogenic substrate for β-galactosidase activity (50, 51) demonstrated that aged lungs (80–82 wk old) had increased frequency of senescent EpCAM^+^ AEC and CD11c+ MHCII^hi^ DC. In contrast, most CD31^+^ endothelial cells were nonsenescent compared with naïve adult lungs (Fig. 3*A*).

Glucocorticoids (GC) such as Dex exerts its immunosuppressive functions via the cytosolic receptor, glucocorticoid receptors (GRs encoded by the *Nr3c1* gene), a member of the nuclear hormone receptor superfamily (52). GR is primarily located in the cytosol and remains inactive in the absence of ligand binding (53); however, it diffuses across the cell membrane and undergoes conformational change following activation (54). Based on the alternative use of exons 9a and 9b, a single GR mRNA can generate functionally distinct two isoforms GRα and GRβ (55). The predominant GRα isoform of the receptor operates as a classical agonist receptor (56), whereas the GRβ lacks the ligand-binding domain and functions as a dominant-negative regulator (57, 58). The elevated level of GRβ suppresses GRα-mediated anti-inflammatory gene activation and is potentially linked to steroid resistance (58, 59). Our results show that GR isoforms expression was reciprocally regulated in lung DCs and airway epithelial cells (AECs) with HDM challenge and Dex treatment in aged mice (Fig. 3*B*). Since the ratio of GRα/GRβ expression was significantly lower in lung DCs than in steroid-responsive AECs with HDM and Dex treatment, these findings strongly suggest an indispensable contribution of aged DC in steroid insensitivity. Senescence-associated secretory phenotype (SASP) such as P16, MMP10, MMP12, and Col1a1 that wield potent effects on adjacent DC, were markedly elevated with aging both in AECs and lung DCs (Fig. 3, *C* and *D*). Collectively, these findings suggest that senescence-associated changes in aged mice may influence DC activity such as antigen uptake and migration to the DLn, thereby influencing airway inflammation and T cell-mediated allergic immune response to the inhaled aeroallergen HDM.

### An Increase in Age Progressively Decreases SPLUNC1 Levels

**Figure 4.**
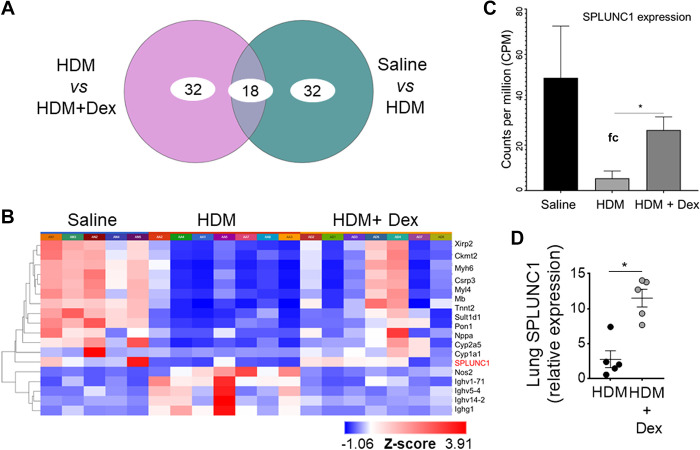
RNA-Seq of HDM-challenged aged mice treated with or without Dex. RNA sequencing data were preprocessed and analyzed using the Picard-STAR-limma pipeline. *A*: Venn diagram shows the top 50 genes that were significantly differentially expressed between no treatment vs. Dex treatment. *B*: using unsupervised hierarchical clustering (HC) analysis based on the differentially expressed genes (DEGs), heatmaps for the top genes among the three groups were generated. Bar graph shows (*C*) three-group comparison of *SPLUNC1/BPIFA1* gene expression using counts per million (CPM) data and (*D*) qRT-PCR of lung SPLUNC1 expression. Data are represented as means ± SE (*n* = 4 or 5 mice per group, *P< 0.01, HDM vs. HDM +Dex, unpaired *t* test). HDM, house-dust mite; SPLUNC1, short palate, lung, and nasal epithelial clone 1.

**Figure 5.**
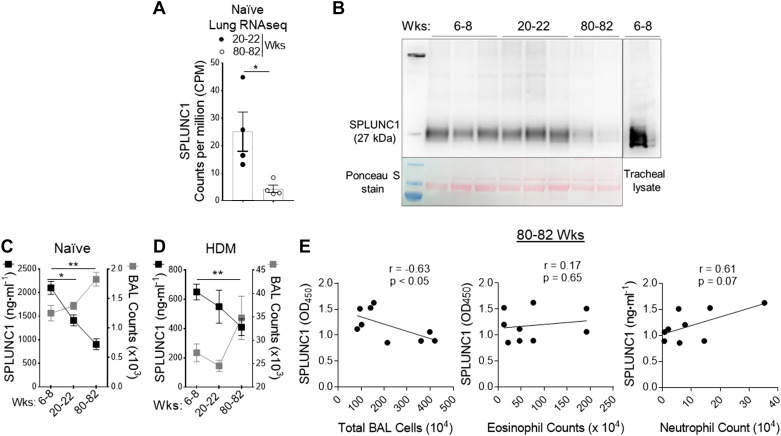
Age-dependent progressive decline of epithelium-derived SPLUNC1 levels. *A*: the bar chart shows counts per million (CPM)-normalized to SPLUNC1 gene expression extracted from naïve adult (20–22 wk old) and aged (80–82 wk old) mice lung RNA-Seq data. *B*: immunoblot showing SPLUNC1 level in BAL from the young, adult, and aged naïve mice; [*upper* lane, SPLUNC1 protein in BAL samples (50 µL/lane), and tracheal lysate; *lower* lane, corresponding Ponceau S staining of the nitrocellulose membrane developed from the same blot]. SPLUNC1 level compared with corresponding total cell counts in BAL from (*C*) naïve and (*D*) HDM-challenged allergic mice as measured by ELISA. *E*: correlations between BAL levels of SPLUNC1 with absolute numbers of total and inflammatory cell types from HDM-inflamed aged lung. Pearson correlation coefficients and associated *P* values are shown for significant relationships only. Results are represented as means ± SE of two independent experiments, *P< 0.05, **P< 0.01. BAL, bronchoalveolar lavage; HDM, house-dust mite; SPLUNC1, short palate, lung, and nasal epithelial clone 1.

We used genome-wide expression profiling of the asthmatic lung transcriptome by RNA-Seq, using the treatment model of HDM-induced asthma in aged mice (Fig. 4) to directly identify lung genes that demonstrate persistently upregulated expression despite Dex therapy during aging. All samples passed stringent quality control criteria. RNA-Seq-based gene expression analysis of aged lungs showed significantly differentially expressed genes with or without Dex treatment (Fig. 4, *A* and *B*). We observed that mRNA expression of SPLUNC1 was significantly modulated with HDM and further by Dex treatment (Fig. 4, *C* and *D*). In addition, we extracted RNA-Seq data of counts per million (CPM)-normalized SPLUNC1 gene expression from naïve mice. Comparison of adult (20–22 wk old) and aged (80–82 wk old) naïve mice revealed a significant decrease in lung SPLUNC1 transcript level in aged as compared with adult mice (Fig. 5*A*).

Furthermore, we detect SPLUNC1 levels in BAL from the young, adult, and aged naïve mice (Fig. 5*B*). As shown in Fig. 5, *C* and *D*, SPLUNC1 levels in BAL were markedly decreased with increasing age and further by HDM-induced airway inflammation. A negative correlation was found between SPLUNC1 protein level and total inflammatory cell numbers in HDM-induced aged mice; however, differential cell analysis reveals a modest association between inflammatory cell types (Fig. 5*E*). Congruently, these results showed that an age-dependent decline in SPLUNC1 level significantly correlated with airway inflammation, suggesting an anti-inflammatory role of SPLINC1 in the aging lung.

### SPLUNC1 Induces DC Activation and Maturation

**Figure 6.**
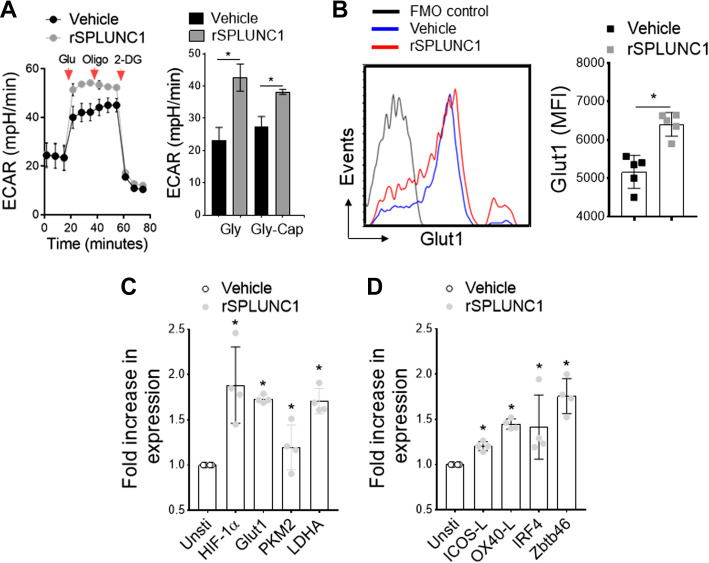
SPLUNC1 induces DC glycolytic metabolism. HDM-pulsed BMDCs (6- to 8-wk-old mice) were stimulated with and without recombinant (r) SPLUNC1 (rSPLUNC1; 5 µg/mL) for 16 h before metabolic measurements. *A*: kinetic extracellular acidification rate (ECAR) in response to glucose (10 mM), oligomycin (2 µM), and 2-DG (100 mM) measuring glycolytic flux/capacity were graphed over time (arrowhead indicates sequential drug injection). Bar graph (*right*) represents glycolysis (Gly) and glycolytic capacity (Gly-Cap) of HDM-pulsed BMDCs in response to rSPLUNC1. Data are represented as means ± SE of two independent experiments. *B*: overlay flow histogram (*left*) and bar graph (*right*) showing mean fluorescence intensity of Glut1^+^ CD11c^+^MHCII^+^CD11b^+^ HDM-pulsed BMDCs treated with rSPLUNC1 and/or vehicle control. *C* and *D*: qRT-PCR of glycolytic genes, DC activation markers, and DC-specific transcription factors. Data are represented as means ± SE (*n* = 4–6, *P < 0.05, unpaired *t* test) of at least two independent experiment. BMDCs, bone marrow-derived dendritic cells; DC, dendritic cell; HDM, house-dust mite; SPLUNC1, short palate, lung, and nasal epithelial clone 1.

We and others have shown that allergen-mediated DC activation results in a striking Warburg-like metabolic shift to glycolysis, which is required for enhanced lysosomal acidification in mature DCs for activation and antigen presentation to T cells (48, 60, 61). To determine the biological function of SPLUNC1 on DC activation and maturation, we used real-time measurement of glycolysis in BMDCs isolated from young mice (6–8 wk old) and stimulated with and without recombinant SPLUNC1 (rSPLUNC1). In the presence of HDM, BMDCs showed typical ECAR changes in response to sequential addition of glucose (Glu), inhibition of mitochondrial ATP synthase by oligomycin (Oligo), and glycolysis inhibition by 2-DG, whereas significantly increased by rSPLUNC1 (5 μg/mL) in glycolysis (Gly) and glycolysis-capacity (Gly-Cap) as measured by XFp seahorse analyzer (Fig. 6*A*). Next, we considered whether rSPLUNC1 stimulation could induce glycolytic markers in HDM-pulsed BMDCs. As shown in Fig. 6*B*, incubation with rSPLUNC1 mediate a significant increase in Glut1 surface expression compared with HDM-pulsed control BMDCs. Furthermore, rSPLUNC1 markedly induces expression of glycolytic-associated genes, including HIF-1α, Glut1, PKM2, and LDHA (Fig. 6*C*), as well as activation/maturation markers (ICOS-L, OX40-L) and DC-specific transcription factors (IRF4, Zbtb46) (Fig. 6*D*). These data demonstrate a novel immunomodulatory function of SPLUNC1, inducing the DC activation/maturation process, thereby influencing allergic airway inflammation.

### SPLUNC1 Modulates the Antigen Presentation Capacity of DC

**Figure 7.**
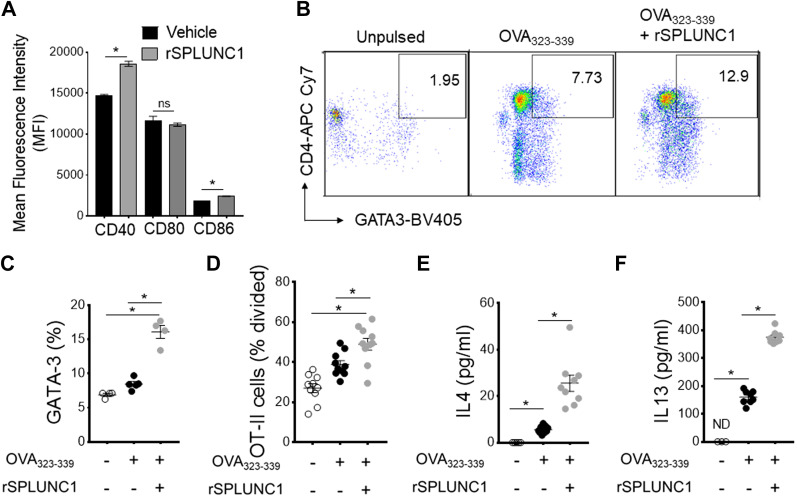
SPLUNC1 modulates the antigen presentation capacity of DC. *A*: mean fluorescence intensity (MFI) of activation markers in CD11c^+^ MHCII^+^CD11b^+^ OVA_323–339_ peptide-pulsed BMDCs stimulated with or without rSPLUNC1 (5 µg/mL). OVA_323–339_ peptide-pulsed BMDCs were incubated (1: 5 ratio) with CFSE-labeled splenic OT-II cells for 4 days, and the effect of rSPLUNC1 on Th2 proliferation and differentiation were enumerated. *B*: pseudocolor plots show illustrative flow cytometry data and (*C*) frequencies of CD4+GATA3+ OT-II cells in coculture. *D*: OVA_323–339_ peptide-specific proliferation was represented as percentage divided OT-II cells and *E* and *F*: Th2 cytokines in coculture supernatant after 4 days were measured by ELISA (*n* = 4–9 per group; *P < 0.01, unpaired *t* test). Data are represented as means ± SE of at least two independent experiments. BMDCs, bone marrow-derived dendritic cells; DC, dendritic cell; SPLUNC1, short palate, lung, and nasal epithelial clone 1.

Since SPLUNC1 induces allergen-induced DC glycolytic reprogramming and maturation, we next hypothesized that SPLUNC1 might interfere with the immune priming capacity of DC to T cells. As shown in Fig. 7*A*, rSPLUNC1 significantly induces the surface expression of maturation markers CD40 and CD86 in HDM-pulsed BMDCs. Coculture experiments of splenic CD4^+^ T cells from mice expressing transgenic MHCII-restricted TCR that recognizes the OVA_323–339_ peptide showed that BMDCs incubated with rSPLUNC1 mediate significant increases in both Th2-specific transcription factor GATA3 expression and T cell proliferation (Fig. 7, *B*–*D*) compared with BMDCs cultured without rSPLUNC1 after ex vivo stimulation with the OVA_323–339_ peptide. Similarly, BMDCs stimulated with rSPLUNC1 showed augmented ability to induce Th2 cytokines, IL13, and IL4 productions in the supernatant following ex vivo stimulation with OVA_323–339_ peptide when cocultured with splenic T cells isolated from OT-II mice as compared with BMDCs culture in the absence of rSPLUNC1 (Fig. 7, *E* and *F*). These experiments collectively show the immune priming capacity of DC by SPLUNC1 to present antigen to CD4+ T cells, thereby promoting the Th2 immune response.

## DISCUSSION

Among the many hallmarks of the aging process (in both mice and humans), is a progressive decline in immune function (immunosenescence) (62, 63). Asthma and its heterogeneity change according to age (4, 64, 65). Asthma is superimposed on a background of aging-associated immune changes in the lung, resulting from complex interactions with various factors such as environmental exposure, microbial triggers, or multiple comorbidities. Asthma in adults is highest among those in middle age; however, mortality is more significant in the older age group (2, 3). According to the World Allergy Organization (WAO), asthma in older adults is phenotypically different from that in young patients, which convolute the diagnosis, assessment, and management in this population (4, 7, 8, 66). Current pharmaceutical interventions to treat asthma in older adults are almost exclusively designed to alleviate symptoms. However, inflammation plays a seminal role in asthma development and progression. A recent clinical trial revealed that mediator-specific asthma controllers such as leukotriene-modifying agents (LTMs) significantly decreased the rate of recurring airway inflammation and are more effective in older adults than inhaled corticosteroids (67–69). The initiation and propagation of airway inflammation arise from several factors, including mediators generated by resident airway cells and recruited leukocytes. Developing a robust T cell response in the lungs requires efficient allergen recognition and activation and migration of lung DCs to the draining DLn, in which the T cell response is primed. Defects in DC function have been identified in some, but not all, studies of older populations (70–72).

Recent studies have detected a very high level of secreted SPLUNC1 (short palate, lung, and nasal epithelial clone1) proteins at the airway (nasal, tracheal, and bronchial) epithelium under the physiological conditions, which act as an airway sensor of innate immunity. SPLUNC1 exerts antimicrobial properties due to structural similarity with the bactericidal/permeability-increasing (BPI) protein fold-containing heterogeneous group of proteins (39, 40). Using *SPLUNC1*^−/−^ mice, previous studies have demonstrated that lack of SPLUNC1 expression in the lung enhances eosinophilic airway inflammation and Th2 allergic asthma in an eotaxin-2-dependent mechanism by alveolar macrophages. However, it is unknown if SPLUNC1 has an immunomodulatory role in regulating the maturation of DC and responses to allergic sensitization impacting airway inflammation. Here, we investigated whether increased age modifies epithelium-derived SPLUNC1 levels and controls DC effector function, thereby bridging lung adaptive T cell-mediated immune response to inhaled allergen. HDM-inflamed lungs of aged mice manifested *1*) decline in pulmonary function; *2*) mixed granulomatous airway inflammation (predominantly of neutrophils and eosinophils), *3*) Th1/Th17-high and Th2-low immune response (which is poorly controlled by Dex treatment) as compared with adult mice. This is consistent with previous findings from people with asthma and mouse models showing allergen-induced eosinophilic inflammation and Th2 immune response that are fully responsive to Dex treatment in adults, whereas allergen-induced neutrophilic inflammation and Th1/Th17 immune response that are refractory to Dex treatment in aging lungs (7, 11, 13, 65).

**Figure 8.**
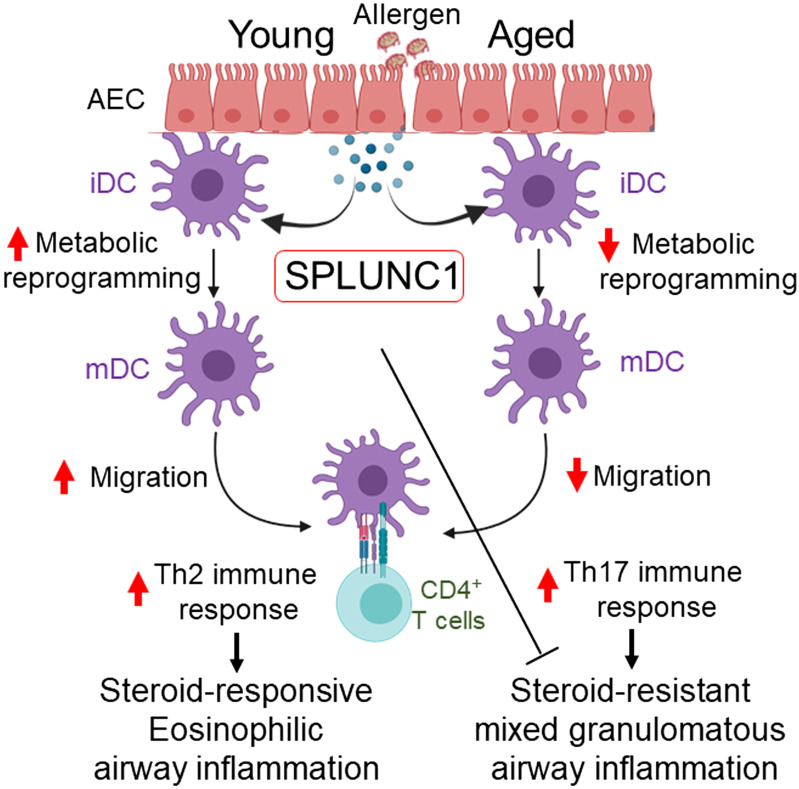
Schematic representation and proposed mechanism of SPLUNC1 and asthma heterogeneity in aging. Increasing age progressively decreases epithelium-derived SPLUNC1 levels, modulating DC effector function and developing dysregulated T cell-mediated immune response to the inhaled allergen, which results in severe airway inflammation and asthma heterogeneity in aging. DC, dendritic cell; iDC, immature DC; mDC, mature DC; SPLUNC1, short palate, lung, and nasal epithelial clone 1.

Interestingly, nasal AECs isolated from subjects with asthma (Severe Asthma Research Program 3 cohort) carrying CC allele expressed less SPLUNC1, which negatively correlated with the severity of the disease (asthma endpoints such as FEV%, fractional exhaled nitric oxide, and serum IgE) as compared with CT and TT genotypes (73). Moreover, IL13-induced increased eotaxin-3 production in AECs by CC genotype is reversed by rSPLUNC1 treatment. In accord with that, our results showed an age-dependent decline in SPLUNC1 level that impacts DC immune priming function and subsequent T cell polarization, thereby resulting in Th17/neutrophilic polarized allergic response and accelerating steroid-resistant severe asthma (Fig. 8).

The lung microenvironment changes with age that compromise DC migration, distribution, and T cell priming ability (74, 75). Within the lung, in the absence of inflammation, the respiratory mucosa has an integrated network of two conventional DC (cDC) subsets (CD103^+^ and CD11b^+^) (45, 76). It has been posited that aging does not change lung DC numbers; however, it alters the effector functions and subset frequencies (28). DC from aged subjects was reported in the semiactivated state with an elevated basal level of NF-κB and proinflammatory cytokines (29). Consistent with these, we found a progressive decrease in frequencies and numbers of HDM-bearing Alexa-647^+^ DC in the DLn as mice aged, suggesting that the intrinsic ability of DC to migrate to DLn is compromised in aged mice. Candidate molecules that might vary in expression and affect DC maturation and migration include epithelium-derived chemokines, cytokines, pleiotropic bioactive lipid, and protein mediators. Congruently, our findings revealed that HDM-induced eosinophilic inflammation and Th2 immunity in adults are fully responsive to Dex treatment and correlated with the SPLUNC1 level. In contrast, we found HDM promotes mixed granulomatous airway inflammation comprising neutrophils in the aged lung, which are unresponsive to Dex treatment and induce SPLUNC1 expression, suggesting an anti-inflammatory function of SPLUNC1 in the aging lung.

Activation of the primary antigen-presenting DC is coupled to rapid glycolytic shift and enhanced lysosomal acidification, which is required for an effective immune-priming effector function (48, 60). We further examined if SPLUNC1 could exert an immunomodulatory effect on DC metabolic reprogramming and immune priming function. Glycolytic reprogramming was assessed in vitro in HDM-pulsed cultured BMDCs treated with recombinant (r) SPLUNC1. Our results reveal that rSPLUNC1 accelerates HDM-induced glycolytic shift and upregulates Glut1 expressions in DC, accompanied by an increase in expression of glycolytic pathway associated genes including *HIF1-α*, *PKM2*, and *LDHA.* Furthermore, in response to rSPLUNC1 stimulation, we observed increased expression of costimulatory molecules, Zbtb46 (cDC-specific zinc finger transcription factor) (77) and IRF4 (78), which are crucial for maturation and Th2 immune priming capacity of DC. In a potential mechanistic link, our results showed enhanced antigen presentation capabilities of OVA_323–339_ peptide-pulsed BMDCs in the presence of rSPLUNC1, as evidenced by increased proliferation of naïve OT-II cells and Th2 effector cytokines IL4 and IL13 production. Collectively, these data indicate that SPLUNC1 modulates DC glycolytic reprogramming and accompanies DC maturation and Th2 differentiation.

SPLUNC1 protein comprises an S18-ENaC-regulatory domain, a central lipid-binding region (tubular lipid-binding proteins), and a C-terminal domain that binds to Ca^2+^ influx channel Orai1 (43, 79). Interestingly, lung DC activation and migration are driven by calcium-activated potassium channel KCa3.1 (80), which could act as a binding partner for epithelial-derived SPLUNC1 proteins regulating DC effector function, and needs to be addressed in the future. Moreover, it is unlikely that a single factor is driving asthma heterogeneity and airway inflammation in aging. Thus, a deeper understanding of the DC-AEC cross talk and how senescence impacts airway inflammation and influences asthma progression are further warranted through state-of-the-art single-cell RNA-Seq (scRNA-Seq) signatures of aging (12, 64).

In conclusion, the present study demonstrates an explanation for asthma heterogeneity and airway inflammation in the aging lung that correlates with SPLUNC1 expression. Moreover, RNA-Seq of aged lungs reveals that SPLUNC1 expression is induced by steroid (Dex) treatment, suggesting an anti-inflammatory function of SPLUNC1, which further influences T cell-mediated adaptive immune responses by modulating effector DC activation/maturation. Nevertheless, the results of our study highlight the causal role of SPLUNC1 expression that may be in part coupled with exaggerated airway inflammation and asthma heterogeneity in aging.

## GRANTS

This study was supported by the Department of Pathobiology, College of Veterinary Medicine, Auburn University and by the National Institute of Allergy and Infectious Diseases (NIAID) of the National Institutes of Health under Award No. R03AI153794-01A1 (to A. Mishra).

## DISCLAIMERS

This article was prepared by A.M. The opinions expressed in this article are the authors’ own and do not reflect the views of the National Institutes of Health, the Department of Health and Human Services, or the United States government.

## DISCLOSURES

No conflicts of interest, financial or otherwise, are declared by the authors.

## AUTHOR CONTRIBUTIONS

A.K.J., J.Y., S.M., M.S., and A.M. performed experiments; A.K.J., J.Y., M.S., A.S., A.K.M., and A.M. analyzed data; A.K.J., J.Y., A.S., and A.M. interpreted results of experiments; A.K.J., J.Y., M.S., A.S., A.K.M., and A.M. prepared figures; A.K.J., J.Y., and A.M. drafted manuscript; A.K.J., J.Y., S.M., and A.M. edited and revised manuscript; A.K.J., J.Y., and A.M. approved final version of manuscript.
